# Biological pathways modulated by antipsychotics in the blood plasma of schizophrenia patients and their association to a clinical response

**DOI:** 10.1038/npjschz.2015.50

**Published:** 2015-12-09

**Authors:** Daniel Martins-de-Souza, Fiorella A Solari, Paul C Guest, René P Zahedi, Johann Steiner

**Affiliations:** 1 Laboratory of Neuroproteomics, Department of Biochemistry and Tissue Biology, Institute of Biology, University of Campinas (UNICAMP), Campinas , Brazil; 2 UNICAMP’s Neurobiology Center, Campinas, Brazil; 3 Leibniz Institut für Analytische Wissenschaften (ISAS), Dortmund, Germany; 4 Department of Psychiatry and Psychotherapy, University of Magdeburg, Magdeburg, Germany; 5 The Center for Behavioral Brain Sciences, Magdeburg, Germany

## Abstract

Proteomics is a valuable tool to unravel molecular mechanisms involved in human disorders. Considering the mediocre effectiveness of antipsychotics, which are the main class of drug used to treat schizophrenia, we analyzed a cohort of 58 schizophrenia patients who had blood collected before and after 6 weeks of antipsychotic treatment using a shotgun mass spectrometry proteomic profiling approach. Our aim was to unravel molecular pathways involved with an effective drug response. The results showed that all patients had essentially the same biochemical pathways triggered Independent of the antipsychotic response outcome. However, we observed that these pathways were regulated in different directions in blood samples from those who responded well to antipsychotics, compared with those who had a poorer outcome. These data are novel, timely and may help to guide new research efforts in the design of new treatments or medications for schizophrenia based on biologically relevant pathways.

Schizophrenia is the result of a combined dysfunction of genetic, biochemical and neurodevelopmental components that may be triggered by environmental factors. This cascade of events will most likely lead to disruption of cellular and tissue homeostasis, impairing the molecular pathways, which govern the cellular machinery throughout the body. Given the widespread nature of these effects, multiplex molecular approaches such as proteomics are required to provide new insights, as such methods can target hundreds of disease-relevant molecules, including those involved in the pathogenesis. In addition, by identifying specific proteins that are present at different steady-state levels in the disease state, proteomics can aid the discovery of molecular biomarker candidates.^1^

Although our understanding of the molecular basis of schizophrenia has evolved recently, there are still many factors that do not connect or are still unknown. Thus, identification of these factors and increasing our understanding of their impact on the disease would be an important step forward. In addition, no factors emerging from molecular studies have been translated into clinical use for schizophrenia studies to date, despite the urgent need for both disease- and medication-related biomarker candidates. This is of particular importance owing to the fact that schizophrenia is an incurable disorder, which demands continuous healthcare, thus generating lifelong suffering for the patients and substantial expenses on the health-care systems. In the US, treatment and management of schizophrenia was estimated at a cost of 62 billion dollars per year, in the year 2000.^2^

Although psychosocial interventions are available, disease management is mainly based on treatment with antipsychotic medications. However, ~40% of schizophrenia patients do not respond properly to these medications and >60% end up abandoning treatment due to undesirable side effects.^3^ Consequently, the intellectual and cognitive capacities of the patients may worsen, making them incapable of functioning adequately in society, thereby producing a further socioeconomic burden.

Consistent with our lack of understanding of disease pathophysiology, knowledge on the effects of medication on metabolism and other molecular pathways in the patients is scarce. With this in mind, we have employed a proteomic approach in an attempt to increase the understanding of the molecular pathways affected in response to currently used atypical antipsychotic medications. It was of particular interest to identify biomarker candidates associated with a positive response. The availability of such early-response biomarkers could eventually be used to help diminish the duration of poor response periods, thereby diminishing disease severity and improving the outcomes for the patients.

The cohort studied in this investigation consisted of 58 acutely ill patients who received the atypical antipsychotic drugs olanzapine (*n*=18), quetiapine (*n*=14) or risperidone (*n*=26) (Table 1). Blood samples were collected by intravenous puncture as previously described in the psychiatric Clinic of the University of Magdeburg (Germany).^4^ Citrate plasma samples were collected at baseline (T0), when patients were acutely ill and either antipsychotic-naive (*n*=23) or antipsychotic-free for at least 6 weeks (*n*=35), and after 6 weeks when all the subjects had been treated as inpatients (T6). Patients with other medical conditions such as type 2 diabetes mellitus, hypertension, cardiovascular or autoimmune diseases were excluded. In line with previous studies,^5^ the patients were separated into responders (*n*=36) and non-responders (*n*=22), with response defined as a 50% reduction of the corrected (subtraction of minimum scores representing ‘no symptoms’) total Positive and Negative Syndrome Scale (PANSS) scores. The percentage of responders was similar in the antipsychotic-naive (61%; 14/23) and antipsychotic-free (63%; 22/35) subgroups (Table 1).

Blood samples were immediately centrifuged for 10 min at 2,000×*g* and resulting supernatants divided in 250-μl aliquots, which were immediately frozen at −80 °C. Protein concentrations were determined by Bradford assay. Next, the plasma samples were depleted of 14 high-abundant proteins using the MARS-14 immunodepletion system (Agilent; Wokingham, UK)^6^ as these tend to obscure lower abundance proteins in proteomic studies. Flow-through fractions containing mostly low abundant proteins were treated successively with 5 mM dithiothreitol (30 min, room temperature) and 10 mM iodoacetamide (30 min, 60 °C in the dark) to block reactive sulfhydryl groups on the proteins. The proteins in the samples were then enzymatically digested using trypsin (Promega, Heidelberg) at a trypsin:protein ratio of 1:50 for 16 h at 37 °C.

The peptides resulting from the trypsin digestion were analyzed using an Ultimate 3000 Rapid Separation Liquid Chromatography system (Dionex, Amsterdam, The Netherlands) coupled to an Orbitrap Elite mass spectrometer (Thermo Scientific, Bremen, Germany). For the chromatography stage, 1 μg of peptide mixture from each sample was preconcentrated on a 100-μm trapping column (Acclaim C18 PepMap100, 100 μm×2 cm, Thermo Scientific) at a flow rate of 20 μl/min in 0.1% trifluoroacetic acid. This was followed by separation on a 75-μm column (Acclaim C18 PepMap100, 75 μm×50 cm, Thermo Scientific) using a binary gradient of solvents A (0.1% formic acid) and B (0.1% formic acid in 84% acetonitrile) ranging from 3/97% A/B to 58/42% A/B over 160 min at a flow rate of 250 nl/min. In order to minimize carry-over effects from previous sample runs, a wash program with high organic content was used prior to each sample injection.^7^

For the mass spectrometry stage, samples were measured in data dependent acquisition mode, survey scans were acquired in the Orbitrap at resolution 60,000 and the 10 most intense signals were fragmented in the ion trap using collision-induced dissociation (CID). The respective target values and maximum injection times were 10^6^ ions and 100 ms for the MS stage, and 10^4^ ions and 50 ms for the MS/MS stage. Precursor ions with charge states between +2 and +5 were selected for fragmentation, using a dynamic exclusion of 15 s.

The resulting raw data were processed using an in-house version of the MASCOT search engine for protein identification and MASCOT Distiller for label-free spectral counting quantification. The cut-off criteria were set at a minimum of 2 peptides for identification and at least 5 MS/MS spectra for quantification. Differences in protein expression between T6 and T0 samples for each patient were determined using Student's *t-*test (*P*<0.05) with a false discovery rate set at 0.1. Differentially expressed proteins were classified according to their biological and molecular processes using the Human Protein Reference Database (http://www.hprd.org). For interpretation of the biological and biochemical significance of differentially expressed proteins, the UniProt identification codes of proteins with a T6/T0 ratio>2:1 (*P*>0.05) were uploaded into the Ingenuity Pathways Knowledgebase (IPKB). This software determines over-represented pathways by overlaying experimental protein data onto pre-existing biological pathway maps. The shotgun mass spectrometry profiling analysis resulted in identification of 18,227 peptides, corresponding to 985 proteins associated with 10 different biological processes (Figure 1a). Intriguingly, 25% of the proteins are of unknown function and are under investigation by our group.

Samples results were divided in four groups. These were T0 responders (T0-R), T6 responders (T6-R), T0 non-responders (T0-NR) and T6 non-responders (T6-NR), independent of the antipsychotic employed. The comparison T6-R/T0-R resulted in identification of 41 differentially expressed proteins, which ranged across seven biological processes (Table 2). The comparison T6-NR/T0-NR identified 58 differentially expressed proteins, which covered eight biological processes (Table 3). One of the proteins (coronin-2A) was increased in responders and decreased in non-responders. We evaluated the differential expression of coronin-2A by western blot as described previously^8^ in two pools of the same samples analyzed by mass spectrometry to confirm this was not technically biased. The mass spectrometry finding was validated using a primary antibody against coronin-2A (Pierce, PA5–30206) (Figure 1b). The blotting membrane was stained with Ponceau Red and used as loading control.

In general, responders and non-responders showed similar effects on the same biochemical pathways after 6 weeks of antipsychotic treatment (Figure 1c). However, there were some differences as responders showed changes in almost twice the number of proteins involved in protein metabolism pathways, non-responders showed energy metabolism differences, which were not observed in the responders.

Potentially the most important finding of this study was that antipsychotic treatment led to opposite directional changes in some of the component proteins in responders and non-responders, as described for coronin-2A above. For example, the responders showed a 4.1 to 1 ratio of decreased to increased proteins in the pathway ‘regulation of nucleic acids metabolism’, and this ratio was 2.0 to 1 in the non-responders (Figure 1c and d). A similar scenario was observed for altered proteins associated with ‘protein metabolism’. Although most of the protein components of this pathway showed a decrease in responders, all of the proteins of this same pathway were increased in non-responders.

Proteins regulating nucleic acids metabolism modulated by antipsychotics may be pivotal to processes such as DNA methylation and demethylation as well as chromatin remodeling, which may balance gene expression in response to antipsychotics.^9^ The differences we observed here reinforce the notion that the success of antipsychotic medication may be dependent on individual genetic backgrounds. In this scenario, the absorption, transport, receptor binding, metabolism and excretion of antipsychotics will be distinct for each patient. This supports the concept of personalized medicine strategies using pharmacogenomic approaches as a means of maximizing the potential effects of medications.^10^ In this study, antipsychotic treatment also modulated proteins associated with the ‘metabolism of proteins’ pathway oppositely in responders and non-responders. This is most likely related to protein turnover effects and may explain some of the above differences in protein expression levels in the two groups, as described in a previous study of antipsychotic treatment response (Lester *et al.* 2012).

The present results indicate that classifying responders and non-responders on the level of biochemical pathways rather than individual proteins may lead to a better understanding of the diverse effects of antipsychotics in schizophrenia patients. Investigation of individual proteins may not be as useful considering the diverse nature of human samples and the existence of redundant systems for the regulation of biochemical pathways in most biological systems. Nevertheless, several of these proteins such as coronin-2A are currently undergoing further analysis in our laboratory as potential antipsychotic treatment response biomarkers. Besides the added value to strategies of translational and personalized medicine, our results suggest new directions to be taken in the search for more effective treatments and the development of new medications for individuals suffering from schizophrenia.

## Figures and Tables

**Figure 1:**
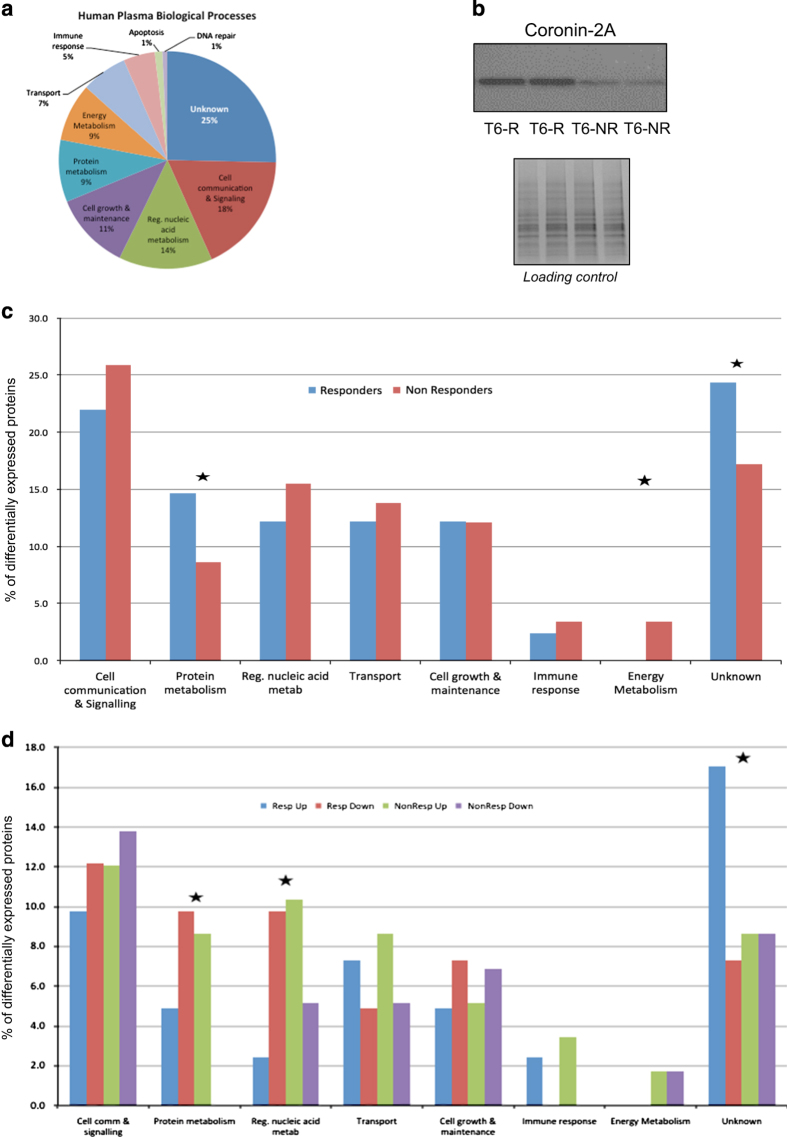
(**a**) Biological processes represented by all 985 plasma proteins identified through the shotgun mass spectrometry profiling approach; (**b**) western blot validation of the mass spectrometry findings for coronin-2A, which showed increased levels in responders and decreased levels in non-responders; (**c**) Biological pathways affected by antipsychotics in responders (blue bars) and non-responders (red bars); (**d**) Percentage of proteins increased in responders (blue), decreased in responders (red), increased in non-responders (green) and decreased in non-responders (purple) in the same biological pathways shown in part (**c**). For (**c** and **d**) the *y* axis represents the percentage of differentially expressed proteins associated to those processes and a star indicates significant differences of the protein constituents (**c**) or directional changes (**d**) within a pathway.

**Table 1:** Patients demographics and PANSS score before and after treatment

*ID patient*	*T0 raw PANSS*				*T0 corrected PANSS*				*T6 raw PANSS*				*T6 corrected PANSS*				*Good response ⩾50% corrected PANSS total reduction*		*First episode patient*	*Drug naive*	*Illness duration [years]*	*Medication*	*Gender*	*Age*	*BMI*	*Smoking*	
	*PANSS P1-P7*	*PANSS N1-N7*	*PANSS G1-G16*	*PANSS total*	*PANSS P1-P7*	*PANSS N1-N7*	*PANSS G1-G16*	*PANSS total*	*PANSS P1-P7*	*PANSS N1-N7*	*PANSS G1-G16*	*PANSS total*	*PANSS P1-P7*	*PANSS N1-N7*	*PANSS G1-G16*	*PANSS total*	*Relative PANSS total improvement T0-T6*	*All patients*									
99	7	17	37	61	0	10	21	31	7	11	29	50	0	4	13	17	0.45	Bad responder	Yes	Yes	0	Quetiapie	F	31	19.7	No	
105	28	32	63	123	21	25	47	93	33	32	64	141	26	25	48	99	−0.06	Bad responder	No	No	15	Quetiapie	M	47	21.8	Yes	
129	22	29	45	101	15	22	29	66	14	24	32	74	7	17	16	40	0.39	Bad responder	No	No		Risperidoe	F	49	23.9	Yes	
133	17	34	49	106	10	27	33	70	10	18	28	59	3	11	12	26	0.63	Good responder	Yes	No	0	Olanzapie	F	25	24.0	Yes	
136	24	24	46	104	17	17	30	64	15	11	26	55	8	4	10	22	0.66	Good responder	No	No		Risperidoe	F	44	27.4	No	
140	25	26	58	115	18	19	42	79	16	15	34	72	9	8	18	35	0.56	Good responder	No	No	7	Risperidoe	M	30	22.2	Yes	
147	31	31	55	126	24	24	39	87	12	24	39	82	5	17	23	45	0.48	Bad responder	Yes	Yes	0	Olanzapie	M	25	28.1	Yes	
151	26	9	40	79	19	2	24	45	8	8	19	38	1	1	3	5	0.89	Good responder	No	No	0	Olanzapie	F	45	30.5	Yes	
156	17	16	28	64	10	9	12	31	12	13	24	54	5	6	8	19	0.39	Bad responder	No	No	2	Risperidoe	M	42	32.9	Yes	
162	20	16	37	76	13	9	21	43	7	14	19	43	0	7	3	10	0.77	Good responder	Yes	Yes	0	Risperidoe	M	19	20.2	Yes	
208	38	18	35	109	31	11	19	61	12	9	20	44	5	2	4	11	0.82	Good responder	No	No	9	Olanzapie	M	28	25.3	Yes	
247	25	25	55	111	18	18	39	75	15	8	27	54	8	1	11	20	0.73	Good responder	No	No	7	Risperidoe	M	40	23.0	Yes	
249	22	41	41	107	15	34	25	74	17	31	37	90	10	24	21	55	0.26	Bad responder	No	No	19	Risperidoe	M	44	17.7	Yes	
260	21	11	31	76	14	4	15	33	8	24	24	59	1	17	8	26	0.21	Bad responder	No	No	1	Olanzapie	M	22	27.5	No	
277	26	12	38	83	19	5	22	46	9	7	21	42	2	0	5	7	0.85	Good responder	Yes	Yes	0	Risperidoe	F	20	22.2	No	
278	37	29	58	137	30	22	42	94	13	22	30	71	6	15	14	35	0.63	Good responder	No	No	9	Risperidoe	M	31	22.8	Yes	
331	31	9	41	92	24	2	25	51	15	8	31	61	8	1	15	24	0.53	Good responder	No	No	23	Olanzapie	F	39	29.8	Yes	
332	23	29	39	95	16	22	23	61	16	24	31	75	9	17	15	41	0.33	Bad responder	Yes	Yes	0	Risperidoe	F	26	22.1	No	
363	27	25	41	100	20	18	25	63	10	16	23	52	3	9	7	19	0.70	Good responder	Yes	Yes	0	Risperidoe	M	25	20.1	Yes	
366	28	16	40	93	21	9	24	54	9	10	21	43	2	3	5	10	0.81	Good responder	Yes	Yes	0	Olanzapie	M	34	27.7	No	
368	34	20	61	132	27	13	45	85	17	20	47	91	10	13	31	54	0.36	Bad responder	Yes	Yes	0	Risperidoe	M	16	26.5	Yes	
370	26	9	39	81	19	2	23	44	9	15	27	54	2	8	11	21	0.52	Good responder	Yes	Yes	0	Risperidoe	M	31	24.4	No	
371	35	14	43	104	28	7	27	62	8	7	20	38	1	0	4	5	0.92	Good responder	No	No	6	Risperidoe	F	48	37.2	No	
375	18	30	50	101	11	23	34	68	21	27	42	97	14	20	26	60	0.12	Bad responder	Yes	Yes	0	Quetiapie	M	23	22.0	Yes	
391	25	11	51	97	18	4	35	57	11	9	22	45	4	2	6	12	0.79	Good responder	No	No	32	Quetiapie	F	55	29.8	No	
400	19	27	54	108	12	20	38	70	7	11	20	41	0	4	4	8	0.89	Good responder	No	No		Quetiapie	F	32	22.7	No	
404	29	10	40	97	22	3	24	49	7	7	20	40	0	0	4	4	0.92	Good responder	No	No	6	Olanzapie	M	45	26.4	Yes	
408	15	13	52	87	8	6	36	50	15	19	41	82	8	12	25	45	0.10	Bad responder	No	No	2	Quetiapie	F	27	22.2	Yes	
410	12	26	47	91	5	19	31	55	20	23	48	98	13	16	32	61	−0.11	Bad responder	No	No		Olanzapie	F	50	24.1	Yes	
422	28	41	45	121	21	34	29	84	7	9	24	43	0	2	8	10	0.88	Good responder	No	No	11	Risperidoe	M	34	23.6	Yes	
426	26	23	46	105	19	16	30	65	12	10	22	47	5	3	6	14	0.78	Good responder	No	No	7	Risperidoe	M	30	21.5	Yes	
428	20	10	60	94	13	3	44	60	19	12	50	89	12	5	34	51	0.15	Bad responder	Yes	Yes	0	Quetiapie	F	38	32.9	Yes	
431	24	26	42	95	17	19	26	62	12	14	26	55	5	7	10	22	0.65	Good responder	Yes	Yes	0	Olanzapie	M	29	21.1	Yes	
440	33	12	45	104	26	5	29	60	13	11	33	65	6	4	17	27	0.55	Good responder	No	No	32	Olanzapie	F	59	35.0	No	
452	14	20	36	80	7	13	20	40	17	9	26	65	10	2	10	22	0.45	Bad responder	No	No	15	Risperidoe	M	36	22.4	No	
454	22	16	48	94	15	9	32	56	7	7	16	33	0	0	0	0	1.00	Good responder	Yes	Yes	0	Risperidoe	M	34	22.2	Yes	
464	25	18	37	94	18	11	21	50	8	14	25	50	1	7	9	17	0.66	Good responder	Yes	Yes	0	Risperidoe	F	66	31.6	Yes	
468	19	18	48	88	12	11	32	55	9	18	24	54	2	11	8	21	0.62	Good responder	No	No	1	Risperidoe	M	35	18.3	No	
474	28	14	51	99	21	7	35	63	32	21	42	105	25	14	26	65	−0.03	Bad responder	No	No	11	Quetiapie	M	32	25.0	Yes	
476	17	17	37	76	10	10	21	41	14	11	32	64	7	4	16	27	0.34	Bad responder	No	No	7	Quetiapie	M	25	22.0	Yes	
479	24	14	44	91	17	7	28	52	13	9	26	52	6	2	10	18	0.65	Good responder	Yes	Yes	0	Olanzapie	F	53	20.1	Yes	
487	37	7	62	115	30	0	46	76	13	17	32	70	6	10	16	32	0.58	Good responder	Yes	Yes	0	Quetiapie	M	39	21.1	Yes	
493	28	29	51	116	21	22	35	78	15	21	29	68	8	14	13	35	0.55	Good responder	Yes	Yes	0	Risperidoe	M	50	19.7	Yes	
520	16	24	38	90	9	17	22	48	9	11	25	49	2	4	9	15	0.69	Good responder	No	No	0	Quetiapie	F	31	28.7	Yes	
521	27	17	50	102	20	10	34	64	10	9	21	43	3	2	5	10	0.84	Good responder	No	No	10	Olanzapie	M	32	30.8	No	
522	17	7	40	67	10	0	24	34	17	9	27	57	10	2	11	23	0.32	Bad responder	Yes	Yes	0	Quetiapie	M	26	21.0	Yes	
524	21	13	40	79	14	6	24	44	12	14	29	58	5	7	13	25	0.43	Bad responder	Yes	Yes	0	Olanzapie	F	36	22.0	Yes	
526	26	38	71	138	19	31	55	105	16	29	39	87	9	22	23	54	0.49	Bad responder	Yes	Yes	0	Olanzapie	M	23	22.2	No	
539	24	27	58	112	17	20	42	79	21	16	49	94	14	9	33	56	0.29	Bad responder	No	No	3	Quetiapie	M	28	23.2	Yes	
541	16	7	30	53	9	0	14	23	13	7	25	56	6	0	9	15	0.35	Bad responder	No	No	34	Risperidoe	M	53	24.8	Yes	
545	28	12	35	84	21	5	19	45	15	7	23	55	8	0	7	15	0.67	Good responder	No	No	0	Olanzapie	F	50	28.6	Yes	
568	21	30	43	100	14	23	27	64	16	26	28	73	9	19	12	40	0.38	Bad responder	No	No	10	Risperidoe	F	56	29.1	No	
570	26	12	45	89	19	5	29	53	11	12	30	56	4	5	14	23	0.57	Good responder	No	No	3	Risperidoe	M	36	20.4	Yes	
572	16	7	25	51	9	0	9	18	7	7	17	34	0	0	1	1	0.94	Good responder	Yes	Yes	0	Quetiapie	F	36	21.9	Yes	
579	14	18	34	75	7	11	18	36	11	7	21	42	4	0	5	9	0.75	Good responder	Yes	Yes	0	Olanzapie	F	36	30.3	Yes	
592	38	25	43	122	31	18	27	76	15	7	41	68	8	0	25	33	0.57	Good responder	No	No	14	Olanzapie	M	30	31.1	No	
663	23	11	22	59	16	4	6	26	10	7	18	38	3	0	2	5	0.81	Good responder	No	No	21	Risperidoe	M	51	30.4	No	
800	16	15	30	65	9	8	14	31	11	8	18	40	4	1	2	7	0.77	Good responder	No	No	15	Risperidoe	M	58	20.6	No	

**Table 2:** Differentially expressed proteins in *responders* before and after 6 weeks of antipsychotic treatment

*Entry*	*Entry name*	*Gene name*	*Score*	*Mass*	*Resp T0×T6*	*Reg*	*StDev*	*Number of peptides*	*Description*	*Biological process*	*Molecular class*	*Molecular function*
Q92828	COR2A_HUMAN	*CORO2A*	35	60,239	0.12	Down	6.913	2	Coronin-2A	Cell communication and signaling	Unclassified	Molecular function unknown
O00622	CYR61_HUMAN	*CYR61*	41	44,165	0.03	Down	1.779	2	Protein CYR61	Cell communication and signaling	Extracellular matrix protein	Extracellular matrix structural constituent
Q5THR3	EFCB6_HUMAN	*EFCAB6*	35	174,702	3.57	Up	7.015	2	EF-hand calcium-binding domain-containing protein 6	Cell communication and signaling	Calcium binding protein	Calcium ion binding
O94887	FARP2_HUMAN	*FARP2*	33	120,725	11.93	Up	3.329	2	FERM, RhoGEF and pleckstrin domain-containing protein 2	Cell communication and signaling	Cytoskeletal protein	Structural constituent of cytoskeleton
P78527	PRKDC_HUMAN	*PRKDC*	40	473,749	41.97	Up	1.948	2	DNA-dependent protein kinase catalytic subunit	Cell communication and signaling	Serine/threonine kinase	Protein serine/threonine kinase activity
Q15036	SNX17_HUMAN	*SNX17*	44	53,153	0.05	Down	3.201	4	Sorting nexin-17	Cell communication and signaling	Adapter molecule	Receptor signaling complex scaffold activity
O43294	TGFI1_HUMAN	*TGFB1I1*	39	51,321	0.31	Down	1.962	2	Transforming growth factor beta-1-induced transcript 1 protein	Cell communication and signaling	Transcription regulatory protein	Transcription regulator activity
Q9UPZ6	THS7A_HUMAN	*THSD7A*	30	192,854	0.03	Down	1.223	2	Thrombospondin type-1 domain-containing protein 7A	Cell communication and signaling	Unclassified	Molecular function unknown
Q6F5E8	LR16C_HUMAN	*RLTPR*	39	156,133	20.44	Up	1.954	2	Leucine-rich repeat-containing protein 16C	Cell communication and signaling	Unclassified	Molecular function unknown
P00740	FA9_HUMAN	*F9*	154	53,114	0.36	Down	6.308	2	Coagulation factor IX	Protein metabolism	Coagulation factor	Extracellular matrix binding
P34931	HS71L_HUMAN	*HSPA1L*	45	70,730	0.45	Down	2.631	4	Heat shock 70 kDa protein 1-like	Protein metabolism	Heat shock protein	Heat shock protein activity
Q06033	ITIH3_HUMAN	*ITIH3*	299	100,072	1.52	Up	1.254	5	Inter-alpha-trypsin inhibitor heavy chain H3	Protein metabolism	Protease inhibitor	Protease inhibitor activity
P09001	RM03_HUMAN	*MRPL3*	28	38,893	0.27	Down	1.813	2	39S ribosomal protein L3, mitochondrial	Protein metabolism	Ribosomal subunit	Structural constituent of ribosome
P01011	AACT_HUMAN	*SERPINA3*	1317	47,792	1.48	Up	1.282	34	Alpha-1-antichymotrypsin	Protein metabolism	Protease inhibitor	Protease inhibitor activity
A1L167	YE019_HUMAN	*UBE2QL1*	71	18,440	0.06	Down	4.273	2	Ubiquitin-conjugating enzyme E2Q-like protein 1	Protein metabolism	Unclassified	Molecular function unknown
Q9UHC1	MLH3_HUMAN	*MLH3*	28	166,060	0.05	Down	2.706	2	DNA mismatch repair protein Mlh3	Reg. nucleic acid metab	DNA repair protein	Protein binding
O75592	MYCB2_HUMAN	*MYCBP2*	35	517,856	6.52	Up	5.641	2	Probable E3 ubiquitin-protein ligase MYCBP2	Reg. nucleic acid metab	Transcription regulatory protein	Transcription regulator activity
Q8IZQ8	MYCD_HUMAN	*MYOCD*	34	102,561	0.43	Down	1.59	2	Myocardin	Reg. nucleic acid metab	Transcription factor	Transcription factor activity
O15350	P73_HUMAN	*TP73*	21	70,206	0.03	Down	1.951	2	Tumor protein p73	Reg. nucleic acid metab	Transcription factor	Transcription factor activity
Q9UL58	ZN215_HUMAN	*ZNF215*	40	60,922	0.11	Down	5.344	2	Zinc finger protein 215	Reg. nucleic acid metab	DNA binding protein	DNA binding
Q2M3G0	ABCB5_HUMAN	*ABCB5*	31	90,173	64.78	Up	1.559	2	ATP-binding cassette sub-family B member 5	Transport	Integral membrane protein	Transporter activity
Q86UQ4	ABCAD_HUMAN	*ABCA13*	70	580,604	12.73	Up	5.368	2	ATP-binding cassette sub-family A member 13	Transport	Transport/cargo protein	Transporter activity
P02768	ALBU_HUMAN	*ALB*	123	71,317	0.04	Down	1.8	6	Serum albumin	Transport	Transport/cargo protein	Transporter activity
P06727	APOA4_HUMAN	*APOA4*	1502	45,371	0.51	Down	1.061	7	Apolipoprotein A-IV	Transport	Transport/cargo protein	Transporter activity
P21796	VDAC1_HUMAN	*VDAC1*	26	30,868	26.82	Up	1.167	2	Voltage-dependent anion-selective channel protein 1	Transport	Voltage-gated channel	Voltage-gated ion channel activity
Q9BXS0	COPA1_HUMAN	*COL25A1*	28	65,145	0.06	Down	2.02	2	Collagen alpha-1(XXV) chain	Cell growth and maintenance	Extracellular matrix protein	Extracellular matrix structural constituent
Q16610	ECM1_HUMAN	*ECM1*	435	62,232	2.43	Up	6.347	2	Extracellular matrix protein 1	Cell growth and maintenance	Extracellular matrix protein	Extracellular matrix structural constituent
Q92556	ELMO1_HUMAN	*ELMO1*	46	84,517	0.13	Down	7.738	5	Engulfment and cell motility protein 1	Cell growth and maintenance	Motor protein	Motor activity
P20929	NEBU_HUMAN	*NEB*	85	775,419	0.08	Down	7.593	6	Nebulin	Cell growth and maintenance	Cytoskeletal protein	Structural constituent of cytoskeleton
P23258	TBG1_HUMAN	*TUBG1*	27	51,480	0.46	Down	2.748	2	Tubulin gamma-1 chain	Cell growth and maintenance	Cytoskeletal protein	Structural constituent of cytoskeleton
O43866	CD5L_HUMAN	*CD5L*	201	39,603	3.03	Up	5.29	2	CD5 antigen-like	Immune response	Secreted polypeptide	Defense/immunity protein activity
Q5JU67	CI117_HUMAN	*C9orf117*	47	60,667	0.02	Down	1.229	2	Uncharacterized protein C9orf117	Unknown	Unclassified	Molecular function unknown
Q8IV32	CCD71_HUMAN	*CCDC71*	26	49,759	32.45	Up	4.607	2	Coiled-coil domain-containing protein 71	Unknown	Unclassified	Molecular function unknown
Q96AQ1	CC74A_HUMAN	*CCDC74A*	31	41,808	12.33	Up	1.079	2	Coiled-coil domain-containing protein 74A	Unknown	Unclassified	Molecular function unknown
Q6IPU0	CENPP_HUMAN	*CENPP*	38	33,429	20.20	Up	1.001	2	Centromere protein P	Unknown	Unclassified	Molecular function unknown
Q6P4F2	ADXL_HUMAN	*FDX1L*	22	19,737	2.01	Up	1.341	2	Adrenodoxin-like protein, mitochondrial	Unknown	Unclassified	Molecular function unknown
Q6ZVN6	YO003_HUMAN	*HSD47*	60	33,173	0.29	Down	1.027	3	Uncharacterized protein HSD47	Unknown	Unclassified	Molecular function unknown
Q8N485	LIX1_HUMAN	*LIX1*	58	32,213	0.23	Down	2.73	5	Protein limb expression 1 homolog	Unknown	Unclassified	Molecular function unknown
Q969T7	5NT3L_HUMAN	*NT5C3B*	40	33,893	9.21	Up	1.238	3	7-methylguanosine phosphate-specific 5'-nucleotidase	Unknown	Unclassified	Molecular function unknown
O15018	PDZD2_HUMAN	*PDZD2*	29	303,964	25.23	Up	4.449	2	PDZ domain-containing protein 2	Unknown	Unclassified	Molecular function unknown
Q7Z6W1	TMCO2_HUMAN	*TMCO2*	22	20,287	2.59	Up	1.144	2	Transmembrane and coiled-coil domain-containing protein 2	Unknown	Integral membrane protein	Molecular function unknown

**Table 3:** Differentially expressed proteins in *non-responders* before and after 6 weeks of antipsychotic treatment

*Entry*	*Entry name*	*Gene name*	*Score*	*Mass*	*Non Resp T0×T6*	*Reg*	*StDev*	*Number of peptides*	*Description*	*Biological process*	*Molecular class*	*Molecular function*
P10645	CMGA_HUMAN	*CHGA*	31	50,829	0.02	Down	1.972	3	Chromogranin-A	Cell communication and signaling	Secreted polypeptide	Defense/immunity protein activity
Q92828	COR2A_HUMAN	*CORO2A*	35	60,239	9.12	Up	1.259	2	Coronin-2A	Cell communication and signaling	Unclassified	Molecular function unknown
O00622	CYR61_HUMAN	*CYR61*	41	44,165	0.42	Down	1.137	2	Protein CYR61	Cell communication and signaling	Extracellular matrix protein	Extracellular matrix structural constituent
Q5THR3	EFCB6_HUMAN	*EFCAB6*	35	174,702	0.01	Down	5.227	2	EF-hand calcium-binding domain-containing protein 6	Cell communication and signaling	Calcium binding protein	Calcium ion binding
O94887	FARP2_HUMAN	*FARP2*	33	120,725	4.14	Up	1.445	2	FERM, RhoGEF and pleckstrin domain-containing protein 2	Cell communication and signaling	Cytoskeletal protein	Structural constituent of cytoskeleton
Q8WXI7	MUC16_HUMAN	*MUC16*	29	2,359,682	0.04	Down	8.085	3	Mucin-16	Cell communication and signaling	Integral membrane protein	Molecular function unknown
Q8NH42	OR4KD_HUMAN	*OR4K13*	33	34,751	0.26	Down	4.954	3	Olfactory receptor 4K13	Cell communication and signaling	G-protein coupled receptor	G-protein coupled receptor activity
P51817	PRKX_HUMAN	*PRKX*	45	41,041	9.97	Up	1.351	4	cAMP-dependent protein kinase catalytic subunit PRKX	Cell communication and signaling	Serine/threonine kinase	Protein serine/threonine kinase activity
Q6ZNA4	RN111_HUMAN	*RNF111*	59	110,119	18.82	Up	1.665	4	E3 ubiquitin-protein ligase Arkadia	Cell communication and signaling	Unclassified	Molecular function unknown
Q16181	SEPT7_HUMAN	*SEPT07_*	29	50,933	4.12	Up	5.889	2	Septin-7	Cell communication and signaling	Cell cycle control protein	Protein binding
Q15036	SNX17_HUMAN	*SNX17*	44	53,153	10.79	Up	1.184	3	Sorting nexin-17	Cell communication and signaling	Adapter molecule	Receptor signaling complex scaffold activity
Q8IWB6	TEX14_HUMAN	*TEX14*	27	169,107	5.62	Up	1.288	2	Inactive serine/threonine-protein kinase TEX14	Cell communication and signaling	Dual specificity kinase	Protein threonine/tyrosine kinase activity
O43294	TGFI1_HUMAN	*TGFB1I1*	39	51,321	0.01	Down	4.21	2	Transforming growth factor beta-1-induced transcript 1 protein	Cell communication and signaling	Transcription regulatory protein	Transcription regulator activity
P30291	WEE1_HUMAN	*WEE1*	51	72,237	0.12	Down	3.019	4	Wee1-like protein kinase	Cell communication and signaling	Dual specificity kinase	Protein threonine/tyrosine kinase activity
Q9UPZ6	THS7A_HUMAN	*THSD7A*	30	192,854	0.01	Down	1.223	2	Thrombospondin type-1 domain-containing protein 7A	Cell communication and signaling	Unclassified	Molecular function unknown
Q9UHC1	MLH3_HUMAN	*MLH3*	28	166,060	0.01	Down	1.087	2	DNA mismatch repair protein Mlh3	Reg. nucleic acid metab	DNA repair protein	Protein binding
Q9UGN5	PARP2_HUMAN	*PARP2*	28	66,734	6.29	Up	1.04	2	Poly [ADP-ribose] polymerase 2	Reg. nucleic acid metab	DNA binding protein	Catalytic activity
Q7L014	DDX46_HUMAN	*DDX46*	117	117,803	8.70	Up	4.669	11	Probable ATP-dependent RNA helicase DDX46	Reg. nucleic acid metab	RNA helicase	Helicase activity
P26358	DNMT1_HUMAN	*DNMT1*	30	185,388	0.07	Down	1.08	2	DNA (cytosine-5)-methyltransferase 1	Reg. nucleic acid metab	DNA methyltransferase	DNA methyltransferase activity
Q8IZQ8	MYCD_HUMAN	*MYOCD*	34	102,561	9.73	Up	2.619	2	Myocardin	Reg. nucleic acid metab	Transcription factor	Transcription factor activity
Q15366	PCBP2_HUMAN	*PCBP2*	17	38,955	0.01	Down	1.318	2	Poly(rC)-binding protein 2	Reg. nucleic acid metab	RNA binding protein	RNA binding
Q5T200	ZC3HD_HUMAN	*ZC3H13*	33	197,203	0.02	Down	2.653	3	Zinc finger CCCH domain-containing protein 13	Reg. nucleic acid metab	Transcription regulatory protein	Transcription regulator activity
Q86UP3	ZFHX4_HUMAN	*ZFHX4*	35	398,157	7.38	Up	7.307	2	Zinc finger homeobox protein 4	Reg. nucleic acid metab	Transcription factor	Transcription factor activity
Q9UL58	ZN215_HUMAN	*ZNF215*	40	60,922	0.02	Down	9.007	2	Zinc finger protein 215	Reg. nucleic acid metab	DNA binding protein	DNA binding
Q86UQ4	ABCAD_HUMAN	*ABCA13*	70	580,604	0.05	Down	1.619	2	ATP-binding cassette sub-family A member 13	Transport	Transport/cargo protein	Transporter activity
P02768	ALBU_HUMAN	*ALB*	123	71,317	0.61	Down	4.848	6	Serum albumin	Transport	Transport/cargo protein	Transporter activity
P06727	APOA4_HUMAN	*APOA4*	1,502	45,371	0.56	Down	1.078	7	Apolipoprotein A-IV	Transport	Transport/cargo protein	Transporter activity
P04114	APOB_HUMAN	*APOB*	1,709	516,666	1.46	Up	1.306	17	Apolipoprotein B-100	Transport	Transport/cargo protein	Transporter activity
Q13439	GOGA4_HUMAN	*GOLGA4*	176	261,892	44.57	Up	1.39	15	Golgin sub-family A member 4	Transport	Transport/cargo protein	Transporter activity
Q9UDX3	S14L4_HUMAN	*SEC14L4*	67	47,070	13.48	Up	1.447	3	SEC14-like protein 4	Transport	Transport/cargo protein	Transporter activity
O14994	SYN3_HUMAN	*SYN3*	34	63,491	0.03	Down	1.434	2	Synapsin-3	Transport	Transport/cargo protein	Transporter activity
P21796	VDAC1_HUMAN	*VDAC1*	26	30,868	8.81	Up	6.002	2	Voltage-dependent anion-selective channel protein 1	Transport	Voltage-gated channel	Voltage-gated ion channel activity
Q96AW1	ECOP_HUMAN	*VOPP1*	32	19,839	2.46	Up	1.841	2	Vesicular, overexpressed in cancer, prosurvival protein 1	Cell growth and maintenance	Transcription regulatory protein	Transcription regulator activity
Q16610	ECM1_HUMAN	*ECM1*	435	62,232	0.31	Down	5.743	2	Extracellular matrix protein 1	Cell growth and maintenance	Extracellular matrix protein	Extracellular matrix structural constituent
Q9P2E2	KIF17_HUMAN	*KIF17*	31	115,784	0.01	Down	1.028	2	Kinesin-like protein KIF17	Cell growth and maintenance	Motor protein	Motor activity
Q9Y496	KIF3A_HUMAN	*KIF3A*	27	80,687	0.01	Down	1.08	2	Kinesin-like protein KIF3A	Cell growth and maintenance	Motor protein	Motor activity
Q14764	MVP_HUMAN	*MVP*	36	99,551	7.09	Up	3.151	4	Major vault protein	Cell growth and maintenance	Transport/cargo protein	Nucleocytoplasmic transporter activity
Q00872	MYPC1_HUMAN	*MYBPC1*	35	129,240	0.01	Down	1.213	2	Myosin-binding protein C, slow-type	Cell growth and maintenance	Structural protein	Structural molecule activity
P23258	TBG1_HUMAN	*TUBG1*	27	51,480	0.02	Down	1.934	2	Tubulin gamma-1 chain	Cell growth and maintenance	Cytoskeletal protein	Structural constituent of cytoskeleton
P02743	SAMP_HUMAN	*APCS*	2577	25,485	1.45	Up	9.908	9	Serum amyloid P-component	Protein metabolism	Secreted polypeptide	Binding
O76031	CLPX_HUMAN	*CLPX*	41	69,922	25.86	Up	2.463	3	ATP-dependent Clp protease ATP-binding subunit clpX-like, mitochondrial	Protein metabolism	Protease	Peptidase activity
P00740	FA9_HUMAN	*F9*	154	53,114	3.06	Up	3.201	2	Coagulation factor IX	Protein metabolism	Coagulation factor	Extracellular matrix binding
P07225	PROS_HUMAN	*PROS1*	56	77,127	2.30	Up	1.065	2	Vitamin K-dependent protein S	Protein metabolism	Coagulation factor	Protease inhibitor activity
A1L167	YE019_HUMAN	*UBE2QL1*	71	18,440	6.16	Up	2.05	2	Ubiquitin-conjugating enzyme E2Q-like protein 1	Protein metabolism	Unclassified	Molecular function unknown
P06681	CO2_HUMAN	*C2*	995	84,583	1.57	Up	1.269	7	Complement C2	Immune response	Complement protein	Complement activity
O14607	UTY_HUMAN	*UTY*	36	151,563	6.28	Up	3.639	4	Histone demethylase UTY	Immune response	Unclassified	Molecular function unknown
Q8WZ42	TITIN_HUMAN	*TTN*	44	3,843,119	4.82	Up	1.558	3	Titin	Energy Metabolism	Structural protein	Structural molecule activity
Q2LD37	K1109_HUMAN	*KIAA1109*	25	559,165	0.01	Down	1.46	2	Uncharacterized protein KIAA1109	Energy Metabolism	Unclassified	Molecular function unknown
Q5JU67	CI117_HUMAN	*C9orf117*	47	60,667	29.92	Up	5.906	2	Uncharacterized protein C9orf117	Unknown	Unclassified	Molecular function unknown
Q8IV32	CCD71_HUMAN	*CCDC71*	26	49,759	0.02	Down	1.543	2	Coiled-coil domain-containing protein 71	Unknown	Unclassified	Molecular function unknown
Q008S8	LFDH_HUMAN	*ECT2L*	39	105,782	0.33	Down	2.308	3	Epithelial cell-transforming sequence 2 oncogene-like	Unknown	Unclassified	Molecular function unknown
Q6P4F2	ADXL_HUMAN	*FDX1L*	22	19,737	5.58	Up	1.218	2	Adrenodoxin-like protein, mitochondrial	Unknown	Unclassified	Molecular function unknown
A6NMB9	FIGL2_HUMAN	*FIGNL2*	25	67,189	0.02	Down	1.825	2	Putative fidgetin-like protein 2	Unknown	Unclassified	Molecular function unknown
Q8WTR4	GDPD5_HUMAN	*GDPD5*	24	69,112	15.84	Up	2.584	2	Glycerophosphodiester phosphodiesterase domain-containing protein 5	Unknown	Unclassified	Molecular function unknown
Q969T7	5NT3L_HUMAN	*NT5C3B*	40	33,893	26.80	Up	2.534	3	7-methylguanosine phosphate-specific 5'-nucleotidase	Unknown	Unclassified	Molecular function unknown
Q68D10	SPT2_HUMAN	*SPTY2D1*	25	75,614	0.09	Down	3.268	2	Protein SPT2 homolog	Unknown	Unclassified	Molecular function unknown
Q7Z6W1	TMCO2_HUMAN	*TMCO2*	22	20,287	6.46	Up	1.541	2	Transmembrane and coiled-coil domain-containing protein 2	Unknown	Integral membrane protein	Molecular function unknown
Q9BZH6	BRWD2_HUMAN	*WDR11*	118	138,423	1.72	Up	3.745	7	WD repeat-containing protein 11	Unknown	Unclassified	Molecular function unknown
